# An evaluation of LSU rDNA D1-D2 sequences for their use in species identification

**DOI:** 10.1186/1742-9994-4-6

**Published:** 2007-02-16

**Authors:** Rainer Sonnenberg, Arne W Nolte, Diethard Tautz

**Affiliations:** 1 Ichthyology, Zoologisches Forschungsmuseum Alexander Koenig, Adenauerallee 160, 53113 Bonn, Germany; 2 Université Laval, Département de Biologie, Laboratoire du Prof. L. Bernatchez, Pavillon Charles-Eugène-Marchand, Ste-Foy, Québec, G1K 7P4, Canada; 3 University of Cologne, Department of Genetics, Zülpicherstrasse 47, 50674 Köln, Germany

## Abstract

**Background:**

Identification of species via DNA sequences is the basis for DNA taxonomy and DNA barcoding. Currently there is a strong focus on using a mitochondrial marker for this purpose, in particular a fragment from the cytochrome oxidase I gene (COI). While there is ample evidence that this marker is indeed suitable across a broad taxonomic range to delineate species, it has also become clear that a complementation by a nuclear marker system could be advantageous. Ribosomal RNA genes could be suitable for this purpose, because of their global occurrence and the possibility to design universal primers. However, it has so far been assumed that these genes are too highly conserved to allow resolution at, or even beyond the species level. On the other hand, it is known that ribosomal gene regions harbour also highly divergent parts. We explore here the information content of two adjacent divergence regions of the large subunit ribosomal gene, the D1-D2 region.

**Results:**

Universal primers were designed to amplify the D1-D2 region from all metazoa. We show that amplification products in the size between 800–1300 bp can be obtained across a broad range of animal taxa, provided some optimizations of the PCR procedure are implemented. Although the ribosomal genes occur in multiple copies in the genomes, we find generally very little intra-individual polymorphism (<< 0.1% on average) indicating that concerted evolution is very effective in most cases. Studies in two fish taxa (genus *Cottus *and genus *Aphyosemion*) show that the D1-D2 LSU sequence can resolve even very closely related species with the same fidelity as COI sequences. In one case we can even show that a mitochondrial transfer must have occurred, since the nuclear sequence confirms the taxonomic assignment, while the mitochondrial sequence would have led to the wrong classification. We have further explored whether hybrids between species can be detected with the nuclear sequence and we show for a test case of natural hybrids among cyprinid fish species (*Alburnus alburnus *and *Rutilus rutilus*) that this is indeed possible.

**Conclusion:**

The D1-D2 LSU region is a suitable marker region for applications in DNA based species identification and should be considered to be routinely used as a marker complementing broad scale studies based on mitochondrial markers.

## Background

The use of DNA sequences as a tool for the identification of species has been widely discussed in the past years [1-6]. One important step in implementing a DNA taxonomy or DNA barcoding system is the choice of appropriate markers. There is currently a preference for the COI gene, a mitochondrial protein, as the marker of choice for animals [1]. But other markers have also been suggested, for example the nuclear rDNA [4,7,8] or the mitochondrial LSU gene (16S rDNA) [9]. It is generally expected that mitochondrial markers could provide a better resolution, because their maternal inheritance results in a smaller effective population size and hence a faster fixation of neutral mutations. However, mitochondrial markers might not always reflect the full history of a species [10]. Nuclear markers, on the other hand, underlie recombination and are therefore less suitable to trace phylogenetic lineages within species.

Apart from the question of choosing the appropriate marker type, there are also general problems with relying on a single marker system only, most notably the possibility of hybridization among lineages. Contrary to a common perception of species being entirely independent from one another, the units that are commonly considered 'species' hybridise frequently in nature [11-14]. Several model systems for interspecific hybridisation have been studied using multiple genetic markers, which suggest that mitochondrial haplotypes and possibly also nuclear genes can be exchanged between distinct species in the course of hybridisation events [15-17]. Even in the absence of hybridisation, incomplete lineage sorting can result in shared ancestral haplotype lineages beyond speciation events [18]. The relevance of these problems in the context of taxonomic applications has been well recognized [2,19], but has so far been very little addressed with respect to developing appropriate comparative markers.

Here we evaluate the utility of the large subunit ribosomal rRNA (LSU) as a potential marker for species identification. The LSU is part of the rDNA gene complex which occurs in tandem repeats, arranged in ribosomal clusters in the nuclear genome [20]. Ribosomal genes are generally considered to be highly conserved, but they are actually composed of a mixture of conserved and divergent regions. These have been called "divergence regions – D" and are numbered in 5'to 3'direction of the mature rRNA [21]. In the present paper we report primers and protocols that permit the amplification of a highly variable part of the nuclear rDNA. We find that the variation in the LSU D1-D2 fragment permits its use in DNA barcoding approaches for a wide variety of metazoa. In addition to this general utility, we explore the use of ribosomal genes to directly identify hybrids, which would not be possible with mitochondrial markers alone.

## Results

### Primer selection

**Figure 1:**
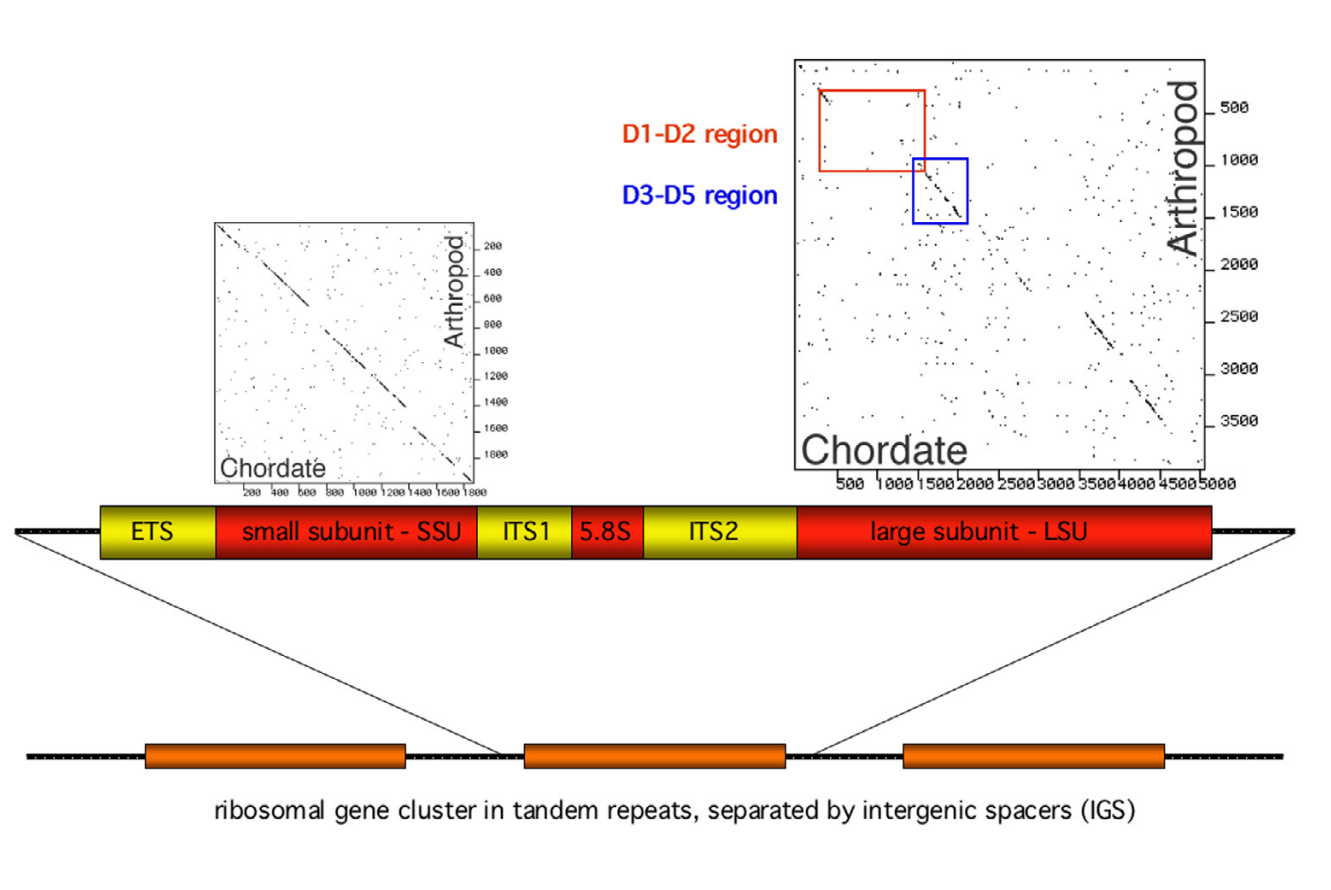
General organisation of eukaryotic ribosomal genes. All eukaryotes show a stereotypic arrangement, with often hundreds of tandemly repeated ribosomal transcription units. Each produces a large transcript which is processed into the small subunit, the 5.8S subunit and the large subunit. The external transcribed spacer (ETS) and the internal transcribed spacers (ITS1 and ITS2) do not become part of the mature rRNA, but their sequences and structures are required for the correct processing. The dot plot comparisons above the SSU and the LSU show the conservation profiles between a chordate and an arthropod sequence. In this presentation, the sequences of two species (mouse and *Drosophila*) are compared and a dot is placed in the diagram at each position where 10 consecutive nucleotides match. Conserved and divergent regions become thus directly apparent. It is evident that the SSU is more conserved, interrupted by a few less conserved regions, while the LSU show larger regions of divergence.

For a survey of meiobenthos organisms we have previously used the D3-D5 expansion segment of the LSU as a signature sequence [7], which has yielded good taxonomic resolution as well as reasonably good phylogenies. However, the fragment includes relatively long highly conserved stretches and we have therefore assessed here the D1-D2 region as an alternative fragment that could potentially yield an even higher resolution. Figure 1 shows a comparison of the relative conservation of the different rRNA regions. It is evident that the D1-D2 region belongs to the most divergent parts, although it contains also some highly conserved stretches that allow the selection of universal primers.

**Figure 2:**
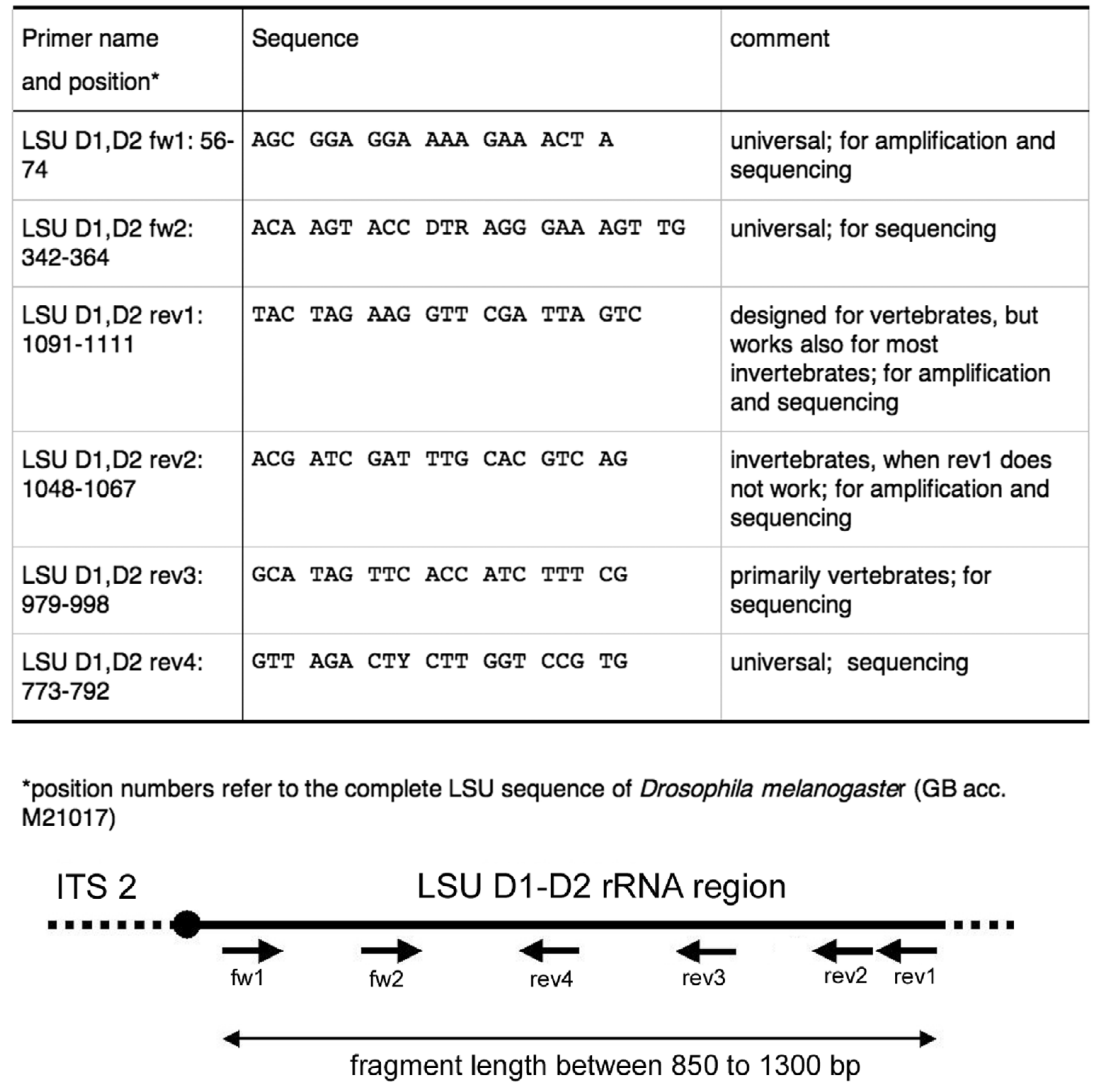
Primers used for amplification and sequencing of the D1-D2 LSU region. The primer positions in column 1 refer to the LSU sequence of *Drosophila melanogaster *(Acc. No. M21017). The sketch below indicates the approximate positions of the primers.

We have retrieved sequences spanning the D1-D2 region of the LSU from diverse metazoan taxa from GenBank and aligned them to identify sequence blocks that are suitable for primer development. Primers were chosen for the most conserved blocks, aiming to identify primers which could be used in very broad range of taxa (with a focus on metazoa, but may also work in all eukaryotes). A few such truly universal primers were identified, but in some cases we had to distinguish between vertebrate and invertebrate primers (Figure 2). The primers were tested on a large range of diverse animal taxa (Table 1) to verify their utility and to find the range of fragment sizes that are to be expected. The outer primers depicted in Figure 2 amplify a fragment of 800–1300 bp, the inner primers can be used for sequencing. Interestingly, the outer primer rev1, which was designed for vertebrates works also with most invertebrates. For those cases where it did not work in invertebrates, we used rev2 as the outer primer.

**Table 1:** List of taxa analysed with observed sequence length range for the D1-D2 fragment in the final alignment [see additional file for further details]

Taxa	number of species	D1-D2 fragment length range (bp)
Teleostei	41°	1041–1186
Cyclostomata	1	1138
Nematoda	17	791–1017
Hirudinea	5	954–1034*
Mollusca	9	986–1005*
Crustacea	5	899–1221*
Chelicerata	1	1066
Insecta	70	945–1302*

* includes sequences amplified with primer rev2, which are in the final alignment 48 bp shorter than those done with rev1.

° without the sequences of *Cottus *(N = 27) and *Aphyosemion *(N = 56) which are within the length range for teleosts.

### Amplification efficiency

We found that the invertebrate sequences could generally be amplified with standard protocols. However, vertebrate samples were generally more difficult to amplify, which cannot only be ascribed to major size differences. Interestingly, DNA traces originating from vertebrate commensals or parasites in DNA extracts were sometimes amplified, while the host sequences were not. We found that residual ribosomal rRNA was a major inhibitor in these cases, since extensive RNAseA digestion of the DNA samples leads to a much better amplification success. Since the same treatment leads also to improved results with invertebrate samples, we have included this in the recommended standard procedure.

Apart of the removal of rRNA we found that the addition of PCR enhancer buffers (see Methods) leads to significantly improved amplification of many rDNA templates, which is in agreement with the observations of Ralser et al. [22]. This is most likely related to high GC content and pronounced secondary structure of our target gene. Finally, single strand binding (SSB)-protein was found to further improve the quality of the PCR product but is not a necessary additive in most reactions.

Further optimizations will still be necessary for some taxa. Most notably, we found that samples from mammals, birds and amphibians are difficult to amplify, i.e. the success rate is low.

### Sequence polymorphisms

**Figure 3:**
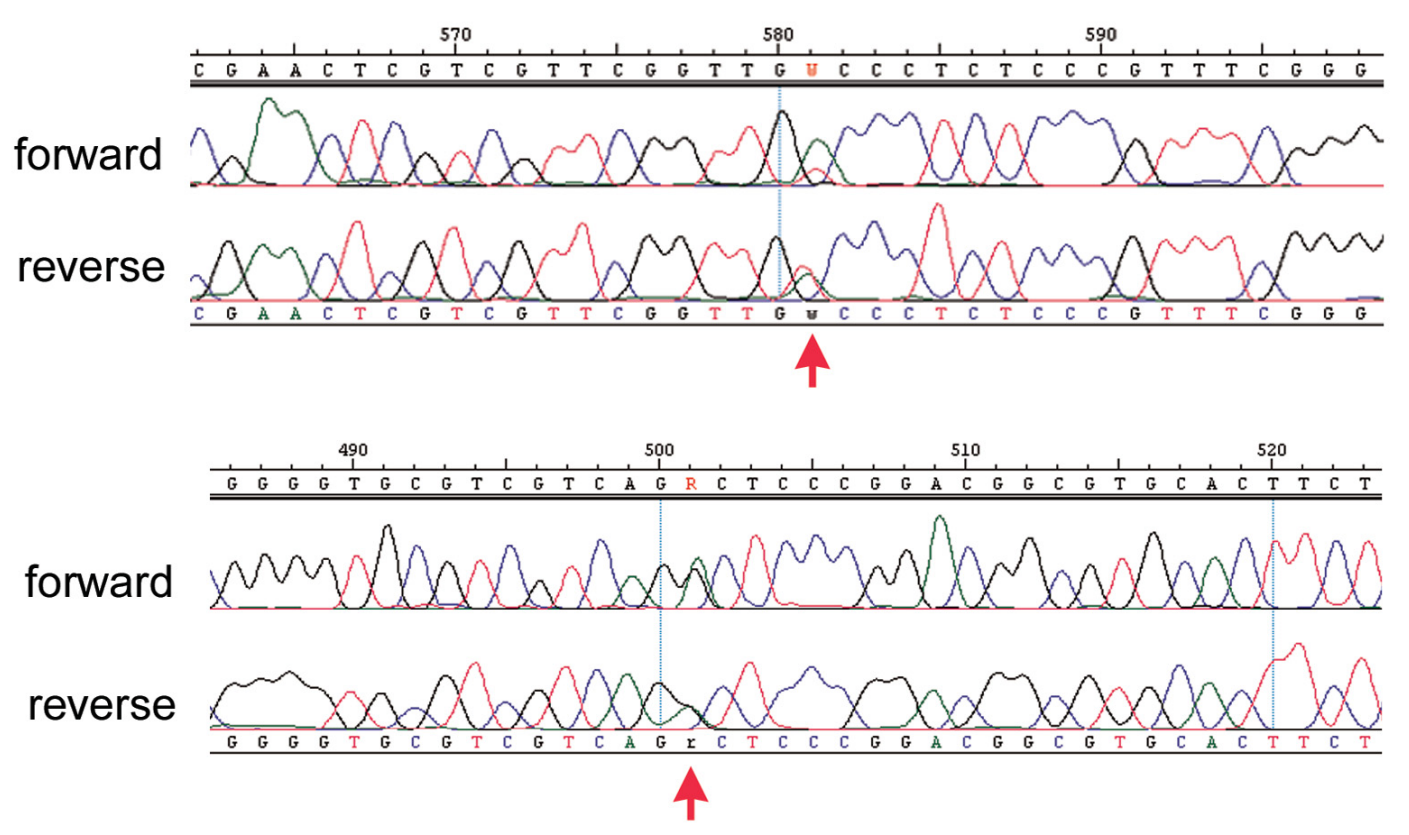
Two examples for ambiguous sequence positions in the forward and reverse sequencing direction of the same fragment (indicated by a red arrow). We interpret these ambiguities as polymorphic positions among the rDNA repeat units within an animal. Given that these ambiguities are very rare (see text), one can conclude that homogenization is usually very efficient.

Ribosomal genes are subject to concerted evolution, which ensures that the multiple copies present in the genome retain more or less identical sequences [23,24]. Still, there may be concerns that the concerted evolution process may not be sufficiently effective to ensure complete sequence homogeneity. We have therefore carefully checked our sequence reads for signs of ambiguity, which would be indicative of incomplete homogenization. Among approx. 230 fragments analysed in this way, we found 15 where a single ambiguous position was identified in both sequencing directions (compare Figure 3), six where two such positions were found and four with three ambiguities. In a few cases we found more ambiguities (up to 9) [see additional file 1], but it is possible that these samples were contaminated with DNA from closely related species (nematodes). This implies that much less than 0.1% of the sequence positions surveyed are polymorphic on average, although this may vary in some taxa. Nonetheless, this number suggests that insufficient homogenization is not a significant problem for D1-D2 sequences.

### Taxon assignment

Information about the affinities of a given sequence was obtained by conducting BLAST searches (blastn) [25] against the GenBank database [26] and by using simple neighbour joining clustering based on uncorrected p-distances with MEGA 3.1 [27]. We found that the phylogenetic signal in the data is sufficient to group most sequences into taxa that are known to be monophyletic, although deep phylogenetic relationships of this tree appeared not to be very reliable (not shown). However, given our focus on a highly variable part of the LSU, we would not have expected to retrieve reliable deep level phylogeny resolution from this anyway. Still, the information content of the D1-D2 region is sufficient to place new sequences into the right phylogenetic context.

### Species identification

**Figure 4:**
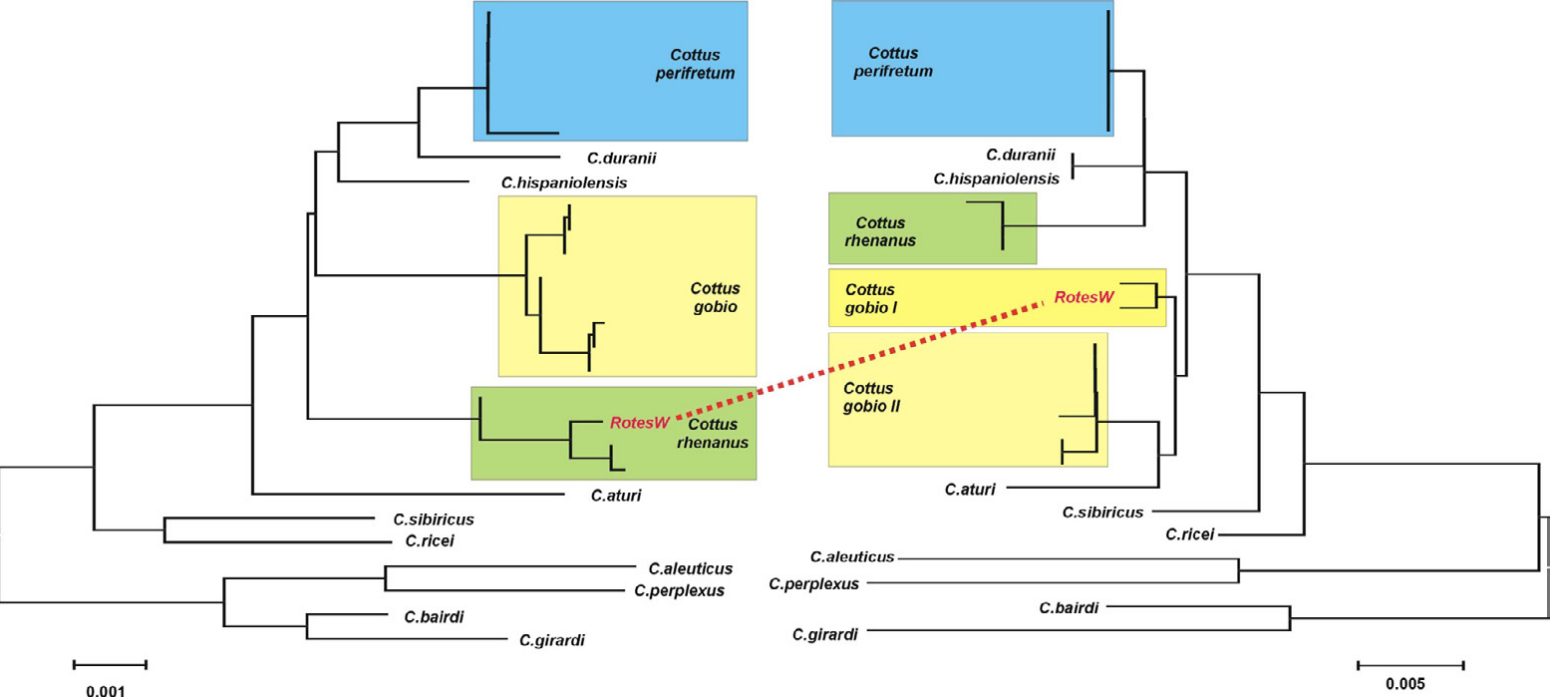
A comparison between LSU and mtDNA sequences with respect to species level resolution for the genus *Cottus*. Twelve species of the genus *Cottus *were sequenced for both markers and trees were obtained via the neighbour joining algorithm in MEGA 3.1. For three species, multiple animals from given populations were sequenced. Both markers detect the same groupings, with the exception of the animals from the population "RotesW", where COI sequences generate a different assignment. Further analysis of this case has shown that this is due to mitochondrial transfer.

The most important consideration for the utility of a marker is its ability to discriminate between closely related species. We have therefore obtained the D1-D2 LSU sequences from two taxonomically well studied fish groups and compared these to mitochondrial COI sequences from the same animals. The first group includes species of the genus *Cottus*, which occur in streams throughout the northern hemisphere. In a recent taxonomic survey of the species in Middle Europe, 15 species were distinguished on the basis of diagnostic morphological and molecular characters [28]. Figure 4 shows a comparison between the LSU sequences and the mitochondrial sequences for a subset of these species, including several population samples from three of them. It is evident that although the general level of divergence is lower for the LSU sequences (note different scales), the same species and the same populations are identified with both markers in most cases. The only exception is the sample "RotesW", which is grouped with *Cottus gobio *for the COI sequence and with *Cottus rhenanus *for the LSU sequence. The origin of the sample, as well as additional molecular markers [15] show that the respective specimen belongs to *Cottus rhenanus *[28], i.e. this is a clear case where a mitochondrial transfer has occurred in the past. The phylogenetic relationships recovered by the two markers show general congruence, but are not identical for all nodes. We have so far no further markers, which could be used to resolve these conflicts, i.e. it is not possible to say whether the LSU or the mtDNA sequences are more reliable in this respect.

**Figure 5:**
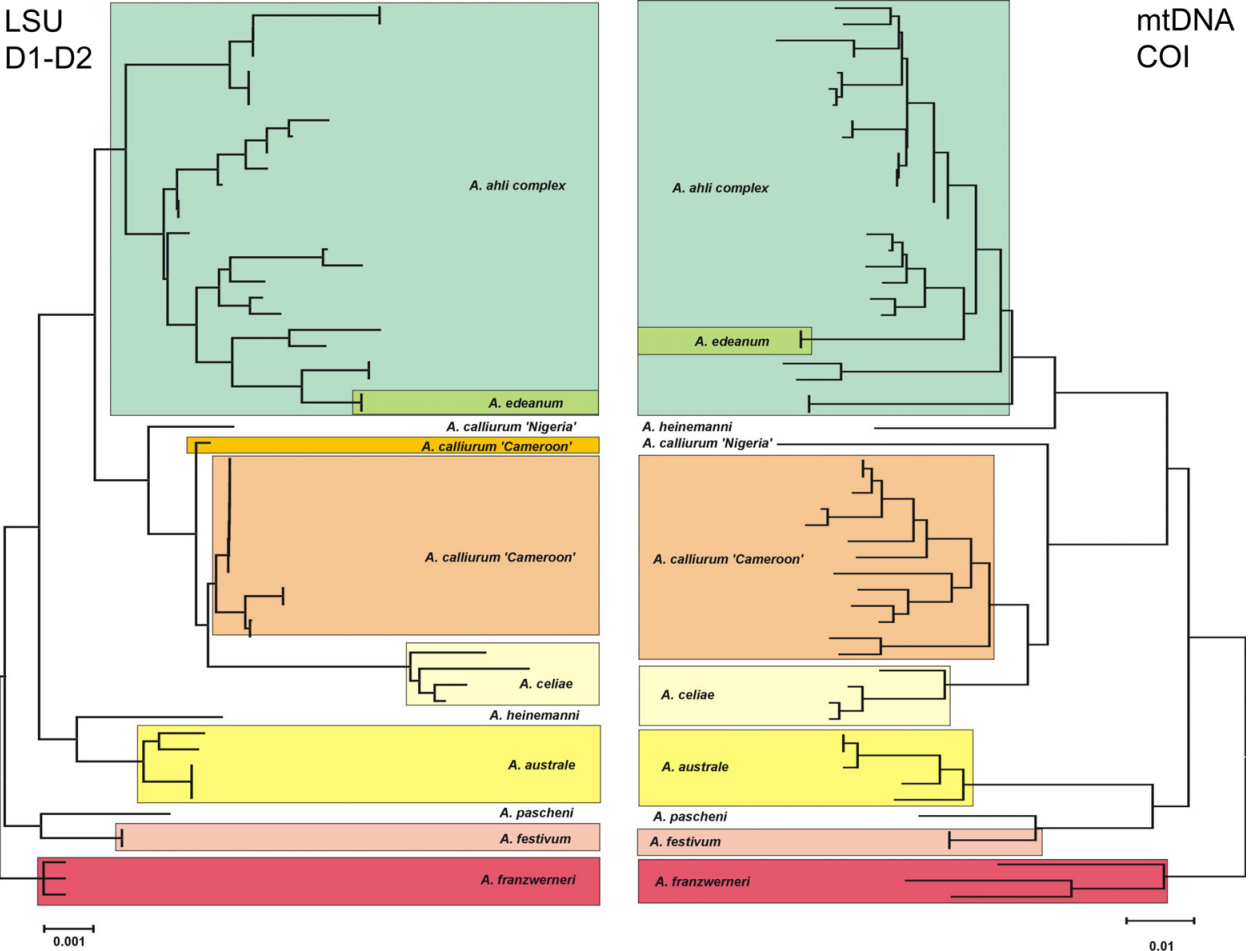
A comparison between LSU and mtDNA sequences with respect to species level resolution for the *Aphyosemion calliurum *species group with a complete taxon sampling. For species with larger distribution several populations were sampled. Both markers assign the samples to the same groups. See text for further details.

The second group is a monophyletic species group (Sonnenberg et al. in prep.) within the large genus *Aphyosemion sensu lato*. These are small fish (ca. 4–5 cm standard length) which live in little forest creeks in the coastal plain from Benin to Cabinda (Angola). Currently nine species and one subspecies are recognized based on diagnostic characters of male colour patterns. Figure 5 shows a comparison between the LSU data and the mitochondrial sequences of all nine recognized species. The sampling contains several populations of species with larger distribution areas (*A. ahli *[Cameroon, Equatorial Guinea], *A. australe *[Gabon to Cabinda (Angola)], *A. calliurum *[Benin to Cameroon]). *A. edeanum*, *A. festivum*, *A. heinemanni *and *A. pascheni *are represented by a single population. Especially the latter three are only known from small areas and all four together with *A. celiae *and *A. franzwerneri *are endemic to Cameroon [29]. Despite a lower sequence divergence level, the LSU dataset shows the same clustering of species samples. *Aphyosemion ahli *and *A. calliurum *are both paraphyletic with respect to *A. edeanum *for the former and *A. celiae *for the latter species. As with the *Cottus *example, there are conflicts with the phylogenetic relationships between species for the two markers (e.g. *A. heinemanni*, *A. australe*) but this depends on the method of phylogenetic inference (NJ, exclusion of gaps and missing data, model of sequence evolution, statistical tests etc.) or insufficient phylogenetic signal and it is still open which of them may be more accurate.

### Evaluation of hybrids

**Figure 6:**
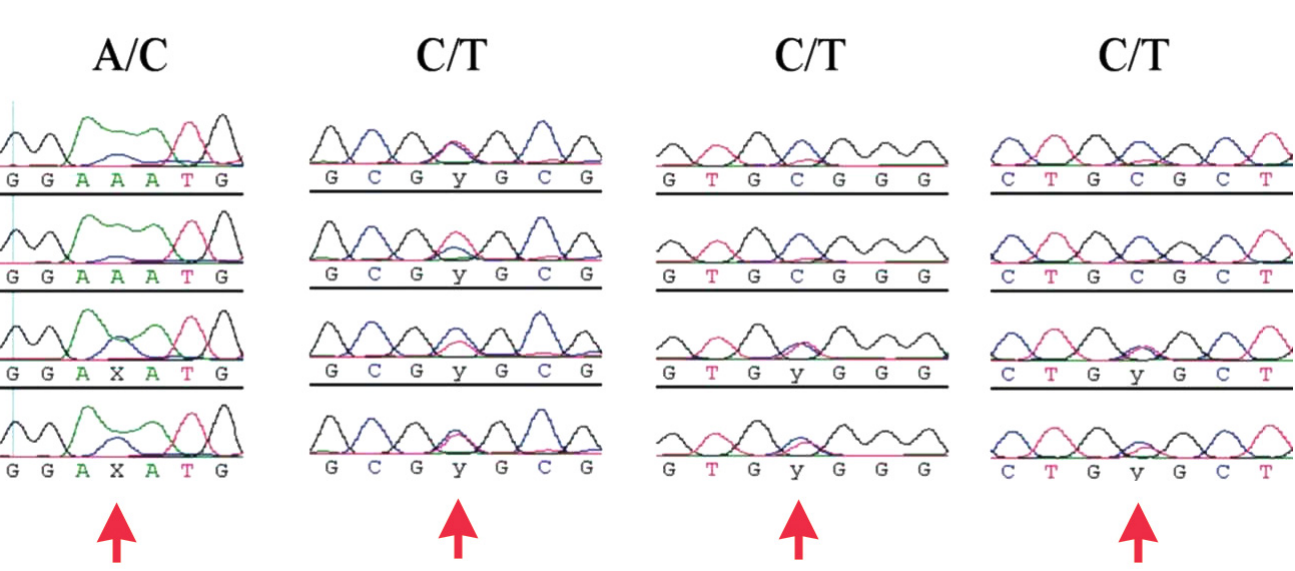
The assessment of hybrid status from heterozygosity of informative characters. The sequence traces were obtained from four F1 hybrid animals between *Cottus perifretum *and *C. rhenanus*. Four positions where fixed differences were known to occur are selected. Double peaks are evident in most, although not in all cases (see text).

To evaluate the performance of the LSU marker in cases of hybrid animals, we have first generated artificial F1 hybrids between *Cottus perifretum *and *Cottus rhenanus*. These two parental species show diagnostic differences in the D1-D2 region. The electropherograms of the hybrid animals were found to display double peaks at these positions (Figure 6), which reflect their hybrid status. Interestingly, however, the height of these double peaks differed between the animals. This suggests that the rDNA repeat units may not be inherited in simple Mendelian patterns, at least not in hybrids between species. Although this presents a complication which needs to be further studied, the presence of such double peaks can indicate a hybrid status, provided one finds the corresponding parent species that have fixed nucleotides at the respective positions.

**Figure 7:**
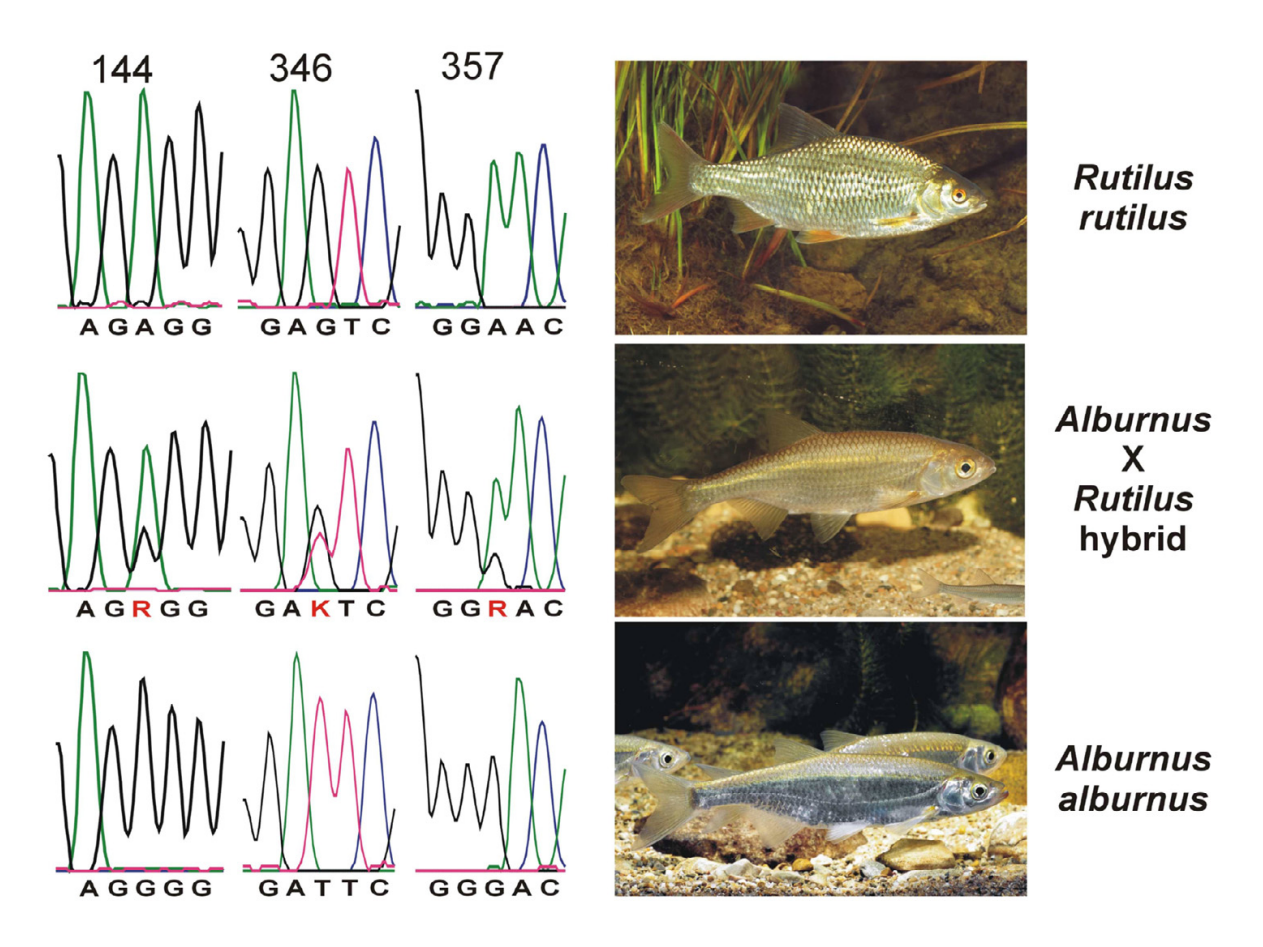
Hybrid status analysis in a natural case. Samples were obtained from pure species of *R. rutilus *and *A. alburnus*, as well as from a suspected hybrid animal. The sequence traces show diagnostic positions for the two species and confirm double peaks at the respective positions for the apparent hybrid animal.

To assess this approach for a naturally occurring situation, we have evaluated it for fish species pair, where the frequent occurrence of F1 hybrid animals has been previously suspected [30]. These are two Cyprinid fish species, *Rutilus rutilus *and *Alburnus alburnus*, which occur in many European streams. Figure 7 shows a comparison of the phenotypes of the parental species and the possible hybrid. The trace pictures of the LSU sequences at three diagnostic sites are shown next to them. It is evident that the suspected hybrid has indeed a mixture of both ribosomal types. This confirms the special utility of rRNA sequences for detecting hybrids.

## Discussion

Our data provide evidence that the D1-D2 region can be readily amplified from a wide range of taxa and that it contains sufficient phylogenetic information to allow assignment to related taxa. At the same time the D1-D2 region appears to carry sufficient variability to distinguish congeneric species although we have so far shown this only for two fish groups. However, at least one of these two groups may be considered as a taxonomic test case because of their close relationships. The *Cottus gobio *complex has previously been considered to represent a single pan-European species. Only a recent taxonomic revision has challenged this view [28,41]. The D1-D2 LSU sequence clusters correspond to the newly described species confirming that a high level resolution is indeed possible with this gene region.

It is noteworthy that much of the variation in the D1-D2 region is found in length variable parts. While such indels cause a problem with alignments for deep phylogenetic inferences, they should be considered as an advantage for taxonomic purposes, since they add another character state beyond transitions and transversions. Moreover, indels may be particularly suitable for fast determination assays independent of sequencing, such as microarrays [7].

### Potential problems associated with the use of ribosomal genes

Ribosomal genes are organized in clusters that contain hundreds of copies per haploid genome. It is generally assumed that these evolve in concert [23,24]. Evidently, intra-genomic variation among these copies could cause problems. However, we have generally observed a low level of ambiguities in our sequences.

Another potential problem in studies of rDNA genes is the occurrence of pseudogenes. Recent studies show that this is a particular problem for the ITS region in animals and plants where it may confound phylogenetic inferences [31,32]. Similarly, multiple variants for the SSU gene were found in sturgeons [33,34], a Platyhelminth [35] and a dinoflagellate [36]. However, both the study by Keller et al. [31] and by Fuerst and Krieger [33] suggest that only the functional variants are expressed at high levels and that these are not polymorphic. Hence, in cases where evidence for pseudogenes is found, it may be useful to directly sequence the ribosomal RNA to assess which of the sequence variants is the functional one.

We note that both problems listed here apply in a similar way also to mitochondrial markers. Insufficient homogenization of rDNA repeats can be compared to heteroplasmy of different mitochondrial haplotypes in a single organism (reviewed in [37]) and rDNA pseudogenes can be compared to nuclear copies of mitochondrial genes (e.g. [38,39]). Thus, for either marker system one has to be aware that complications can arise through these phenomena, although they are not so frequent that they would seriously compromise their broad applicability.

### rDNA variation and taxonomic group delimitation

Any new sequence variant in rDNA has to be homogenized across at least most of the other rDNA repeats before it becomes detectable. This homogenization is most likely achieved by a succession of many unequal cross-over events within the rDNA cluster. Hence, new alleles should only become visible after some time of genetic exchange has passed in an interbreeding population. However, it is currently not clear how long it takes to fix a new variant, or even whether this occurs predominantly via intra- or interchromosomal homogenization [40]. But in any case, a new fixed variant can be seen as a reflection of an extended history of interbreeding in a given population. Accordingly, even a single mutational difference in rDNA may be taken as an informative character that could potentially delineate a new group.

For mitochondrial DNA analysis it was suggested that a threshold level of intraspecies versus interspecies variance is used as a proxy to identify groups or species [1]. However, species recognition (as well as the underlying concepts) is not only an issue of distances but also of distinctness (e.g. [41]). Taxonomic resolution in the context of alpha taxonomy is either present or not and is basically given once a single diagnostic character can be identified. In the case of *Cottus*, we found that the different rDNA variants depicted in Fig. 4 correspond to animals from different streams and that one usually finds only one variant per stream, at least in the cases where the respective data are available. Thus, a separate group recognition based on molecular differences as small as a single substitution is corroborated by their allopatric occurrence and their presumably independent evolution since several thousand years. Accordingly, single nucleotide differences in rDNA genes may be considered as a first indicator of genetic separation. In cases where the respective groups occur under sympatric conditions, it may even be an indicator of incipient speciation. Clearly, this issue has to be further explored, but these considerations show that the collection of sequences from the coding parts of the ribosomal genes may provide an additional value for taxon delimitation.

## Conclusion

Our results show that the D1-D2 region of the LSU rDNA gene has the potential to be developed as a taxonomic marker. It can be amplified with truly universal primers and shows a divergence rate, which is suitable to differentiate even closely related species. Because it is bi-parentally inherited, it can also be used for detecting hybrids and their corresponding parental species. It appears therefore that it could be an ideal marker for complementing DNA barcoding studies based on mitochondrial COI sequences.

## Materials and methods

Macrozoobenthos invertebrates were collected and determined by M. Hess (Munich), nematodes were obtained as cultures from E. Schierenberg (Cologne), most fish material and some invertebrates were collected and/or determined by AN and RS, the *Galaxias *and *Brachygalaxias *samples by K. Busse (Bonn). F_1 _Hybrids of *Cottus *were produced in the aquarium as described in Stemshorn et al. [42].

For most samples total DNA was extracted from ethanol preserved or fresh material using a standard Proteinase-K in SDS/EDTA buffer [tissue digestion in 500 μL HOM buffer (0.5% SDS, 100 mM Tris-HCl, 80 mM EDTA pH 8.0) and 5 μL Proteinase-K (20 mg/mL) for at least 3 h at 55°C; addition of 500 μL NaCl (4.5 M) and 300 μL Chloroform, gentle mixing for 15 min.; centrifugation for 10 min. at 10.000 rpm, transfer of upper phase (750 μL) without interphase in new tube; precipitation with 750 μL 99% Ethanol, gentle mixing and incubation at room temperature for 5 min., centrifugation for 10 min. at 13.000 rpm, removal of supernatant; 2× washing of the pellet with 500 μL 70% Ethanol, incubation at room temperature for 5 min., centrifugation for 10 min. at 13.000 rpm, complete removal of supernatant; airdried pellet dissolved in 100 μL TE buffer (10 mM Tris-HCl, 0.1 mM EDTA pH8.0)]. Alternatively, we used a standard CTAB buffer protocol [tissue digestion in 500 μL 2% CTAB buffer (2 g/100 mL CTAB, 1.4 M NaCl, 100 mM Tris-HCl, 20 mM EDTA, pH 8.0) and 15 μL Proteinase-K (20 mg/mL) for 1 h – overnight at 64°C; 2× extraction with Chloroform/Isoamylalcohol (24:1), gentle mixing for 10 min., centrifugation for 10 min. at 13.000 rpm, transfer of upper phase without interphase in new tube; precipitation with 400 μL 98% Ethanol, incubation at room temperature for 1 h, centrifugation for 20 min. at 13.000 rpm, removal of supernatant; 2× washing of the pellet with 500 μL 75% Ethanol, centrifugation for 10 min. at 13.000 rpm, complete removal of supernatant; airdried pellet solved in 50–100 μL TE]. For some samples we used also DNA isolated with a commercial kit (procedure according to the manufacturer; Qiagen, Düsseldorf) or released the DNA with a Chelex/Proteinase-K protocol (500 μL 5% Chelex suspension, 10 μl Proteinase K (20 mg/ml]) incubation 1 h to overnight at 64°C, 15 min. 95°C denaturation of Proteinase-K (important for the following RNAseA treatment). RNA was digested for all samples with RNAseA (10 mg/ml, Fermentas) before PCR reactions. We added to 50 μL DNA solution 2 μl Fermentas RNAseA and incubated for 1–3 h at room temperature.

Primers were designed according to partial or complete LSU rDNA sequences from GenBank for a variety of taxa, ranging from plathelminths, nematodes and arthropods to vertebrates. Primer sequences are listed in Figure 2.

PCR conditions were tested with temperature and MgCl_2 _gradients. Amplification of LSU fragments were considerably enhanced by the addition of Q-solution (Qiagen, Düsseldorf) and single strand binding Protein (SSB, Sigma Aldrich). Final concentration of Q-solution is 1× (from 5× stock) and 1 μg SSB protein in a 20 μl PCR reaction mix.

The following PCR program was used to amplify the D1-D2 fragments: 4 min. at 94°C for initial denaturation; 45 cycles with 20 sec. 94°C, 20 sec. 52,5°C and 90 sec. 72°C, followed by 8 min. at 72°C for final extension. For most fragments we used the primer combination fw1 and rev1, some invertebrates amplified better with the combination fw1 and rev2.

In addition we amplified and sequenced a COI fragment currently applied in DNA barcoding applications for a sample of *Cottus *and the species of the *Aphyosemion calliurum *group (Cyprinodontiformes: Nothobranchiidae) for comparison with the LSU fragment. PCR primer for the amplification of the COI fragment for these taxa were taken from the literature (HCO-2198 [1,43]) or designed according to published complete mitochondrial sequences and own data for the *Cottus *samples and the *A. calliurum *group. The following primers were used : *Cottus *COI forward: 5'-TTC TCG ACT AAT CAC AAA GAC ATT-3, *Cottus *COI reverse: 5'-TAG ACT TCA GGG TGA CCA AAG AAT CA-3, *Aphyosemion *forward: 5'-TAA GAA AAG GAT TTA AAC CT-3': "universal" reverse[43]: 5'-TAA ACT TCA GGG TGA CCA AAA AAT CA-3'.

All *Aphyosemion *and *Cottus *COI PCR reactions are done in 15 microliter reactions with the Qiagen Multiplex PCR Kit, including 3 microliter Q-Solution and 0.5 microliter of a 10 pmol solution of each primer. The following PCR program was used to amplify the COI fragments: 15 min. 95°C for initial denaturation and activation of the polymerase enzyme; 45 cycles with 20 sec. 94°C, 90 sec. 52°C and 90 sec. 72°C, followed by 8 min. at 72°C for final extension.

PCR products were checked on a 1.5 % agarose gel with ethidium bromide staining (130 V, 30–40 min.), cleaned with Millipore PCR cleaning plates and sequenced according to the manual with ABI BigDye Terminator ver.3.1 in both directions on an ABI 3700. Sequencing was done with the same primers as used in the PCR reaction for both gene fragments. Very long LSU sequences (> 1100 bp), especially if they contain GC rich stretches, were sequenced in addition with the internal primers fw2 and rev4. For sequencing it sometimes turned out to be helpful to increase the amount of template DNA to get better reads in difficult sequence regions.

Contigs were assembled with Lasergene SeqMan II (DNA-Star) and resulting sequences checked against GenBank for contamination. All contigs were checked by eye for ambiguous nucleotides in the regions sequenced for both strands. We counted positions with double peaks from one third up to same height in both strands to estimate the occurrence of different alleles or copies in the rDNA cluster.

The COI and LSU sequences for *Cottus *and *Aphyosemion *were aligned with Clustal X [44] and checked by eye with BioEdit 5.0.9 [45]. Aligned protein sequences were checked for a functional coding sequence to test against non-functional nuclear copies. A cluster analysis was done with the neighbour joining algorithm (NJ) as implemented in MEGA 3.1 [27]. We employed no model of sequence evolution and used p-distances to compare only the raw data without any assumptions on sequence evolution. Missing data or gaps were deleted in the pairwise comparison.

All sequences are deposited in Genbank under the accession numbers EF416965 – EF417284).

## Supplementary Material

Additional File 1 Supplemental list of species. List of all sequences obtained, including species assignment, length, accession number and number of ambiguities found. Click here for file
