# Supplementary material for: An evaluation of LSU rDNA D1-D2 sequences for their use in species identification
Source: Front Zool. 2007 Feb 16;4:6. doi: 10.1186/1742-9994-4-6 (PMC1805435; doi:10.1186/1742-9994-4-6)
Supplement: Additional File 1 — Supplemental list of species. List of all sequences obtained, including species assignment, length, accession number and number of ambiguities found. [file 1742-9994-4-6-S1.pdf]

Supplemental list of species

Cottus dataset

species	no. of ambiguities	taxgroup I	taxgroup II	LSU fragment length (bp)	remarks	GB acc.no. LSU	GB acc.no. COX
Cottus aleuticus		Teleostei	Vertebrata	1030	#	EF417196	EF416965
Cottus aturi		Teleostei	Vertebrata	1030	#	EF417197	EF416966
Cottus bairdi		Teleostei	Vertebrata	1030	#	EF417198	EF416967
Cottus durani		Teleostei	Vertebrata	1030	#	EF417199	EF416968
Cottus girardi		Teleostei	Vertebrata	1030	#	EF417200	EF416968
Cottus gobio (7x)		Teleostei	Vertebrata	981° -1030	#	EF417201 - EF417207	EF416970 - EF416976
Cottus hispaniolensis		Teleostei	Vertebrata	1030	#	EF417208	EF416977
Cottus perifretum (6x)		Teleostei	Vertebrata	1016°-1030	#	EF417209 - EF417214	EF416978 - EF416983
Cottus perplexus		Teleostei	Vertebrata	1031	#	EF417215	EF416984
Cottus rhenanus (4x)		Teleostei	Vertebrata	981°-1030	#	EF417216 - EF417219	EF416985 - EF416988
Cottus ricei		Teleostei	Vertebrata	991°	#	EF417220	EF416989
Cottus sibiricus		Teleostei	Vertebrata	1030	#	EF417221	EF416990

Aphyosemion dataset

species	no. of ambiguities	taxgroup I	taxgroup II	LSU fragment length (bp)		GB acc.no. LSU	GB acc.no. COX
Aphyosemion ahli (24x)	1	Teleostei	Vertebrata	1169-1172	#	EF417222 - EF417245	EF416991 - EF417014
Aphyosemion australe (5x)		Teleostei	Vertebrata	1169-1175	#	EF417249 - EF417253	EF417015 - EF417019
Aphyosemion calliurum (14x)	1	Teleostei	Vertebrata	1166-1170	#	EF417254 - EF417267	EF417020 - EF417033
Aphyosemion celiae (4x)	1	Teleostei	Vertebrata	1186-1192	#	EF417268 - EF417271	EF417034 - EF417037
Aphyosemion edeanum (2x)		Teleostei	Vertebrata	1169	#	EF417246 - EF417247	EF417038 - EF417039
Aphyosemion festivum (2x)		Teleostei	Vertebrata	1179	#	EF417272 - EF417273	EF417040 - EF417041
Aphyosemion franzwerneri (3x)		Teleostei	Vertebrata	1166	#	EF417274 - EF417276	EF417042 - EF417044
Aphyosemion heinemanni		Teleostei	Vertebrata	1170	#	EF417248	EF417045
Aphyosemion pascheni		Teleostei	Vertebrata	1183	#	EF417277	EF417046

Metazoan sequence-data

species	no. of ambiguities	taxgroup I	taxgroup II	LSU fragment length (bp)		GB acc.no. LSU
Dina punctata		Hirudinea	Annelida	982	rev2	EF417047
Erpobdella octoculata		Hirudinea	Annelida	1028		EF417048
Erpobdella vilnensis		Hirudinea	Annelida	1034		EF417049
Helobdella stagnalis		Hirudinea	Annelida	1003		EF417050
Theromyzon tessulatum		Hirudinea	Annelida	954	rev2	EF417051
Avicularia versicolor		Araneae	Chelicerata	1066		EF417052
Lampetra planeria		Agnatha	Chordata	1138		EF417053
Artemia sp.		Anostraca	Crustacea	928		EF417054
Branchipus schaefferi		Anostraca	Crustacea	899	rev2	EF417055
Atyaephyra desmaresti	1	Decapoda	Crustacea	1048		EF417056
Asellus aquaticus		Isopoda	Crustacea	991		EF417057
Porocephalus sp.	2	Pentastomida	Crustacea	1221		EF417058
Agabus sturmii		Coleoptera	Insecta	1058		EF417059
Anacaena lutescens		Coleoptera	Insecta	1051		EF417060
Elodes marginata		Coleoptera	Insecta	1136		EF417061
Gyrinus substriatus		Coleoptera	Insecta	1241		EF417062
Haliplus flavicollis		Coleoptera	Insecta	1142		EF417063
Haliplus heydeni		Coleoptera	Insecta	1074		EF417064
Haliplus immaculatus		Coleoptera	Insecta	1074		EF417065
Hydaticus seminiger		Coleoptera	Insecta	1009	rev2	EF417066
Hydrobius fuscipes		Coleoptera	Insecta	992	rev2	EF417067
Laccobius striatulus		Coleoptera	Insecta	1038		EF417068
Limnius perrisi		Coleoptera	Insecta	1006	rev2	EF417069
Limnius volckmari		Coleoptera	Insecta	1053		EF417070
Orectochilus villosus		Coleoptera	Insecta	1232		EF417071
Platambus maculatus		Coleoptera	Insecta	1042	rev2	EF417072
Liponeura cinerascens minor	1	Diptera	Insecta	1067		EF417073
Oplodontha viridula		Diptera	Insecta	981		EF417074
Simulium reptans	2	Diptera	Insecta	946	rev2	EF417075
Simulium variegatum	3	Diptera	Insecta	945	rev2	EF417076
Acheta domesticus		Ensifera	Insecta	1296		EF417077
Gryllidae sp.	1	Ensifera	Insecta	1302		EF417078
Baetis rhodani		Ephemeroptera	Insecta	1065		EF417079
Caenis robusta		Ephemeroptera	Insecta	1067		EF417080
Cloeon dipterum		Ephemeroptera	Insecta	1075		EF417081
Ecdyonurus insignis		Ephemeroptera	Insecta	1059		EF417082
Ephemerella notata		Ephemeroptera	Insecta	1084		EF417083
Habroleptoides confusa		Ephemeroptera	Insecta	1073		EF417084
Heptagenia sulphurea		Ephemeroptera	Insecta	1082		EF417085
Paraleptophlebia submarginata	1	Ephemeroptera	Insecta	1070		EF417086
Potamanthus luteus		Ephemeroptera	Insecta	1066		EF417087
Seratella ignita		Ephemeroptera	Insecta	1090		EF417088
Torleya major		Ephemeroptera	Insecta	1041	rev2	EF417089
Aphelocheirus aestivalis		Heteroptera	Insecta	1083		EF417090
Aquarius najas		Heteroptera	Insecta	1043	rev2	EF417091
Aquarius paludum		Heteroptera	Insecta	1091		EF417092
Cymatia coleoptrata		Heteroptera	Insecta	1078		EF417093
Gerris lacustris		Heteroptera	Insecta	1090		EF417094
Hydrometra stagnorum	3	Heteroptera	Insecta	1109		EF417095
Ilyocoris cimicoides	3	Heteroptera	Insecta	1036	rev2	EF417096
Micronecta scholtzi		Heteroptera	Insecta	1090		EF417097
Nepa cinerea		Heteroptera	Insecta	1097		EF417098
Notonecta glauca		Heteroptera	Insecta	1082		EF417099
Ranatra linearis		Heteroptera	Insecta	1099		EF417100
Sigara falleni		Heteroptera	Insecta	1084		EF417101
Panorpa communis	1	Mecoptera	Insecta	1069		EF417102
Sialis lutaria		Megaloptera	Insecta	1180		EF417103
Osmylus fulvicephalus		Neuroptera	Insecta	1360		EF417104
Sisyra nigra		Neuroptera	Insecta	1261		EF417105
Calopteryx splendens		Odonata	Insecta	1098		EF417106
Erythromma najas		Odonata	Insecta	1049	rev2	EF417107
Onychogomphus forcipatus		Odonata	Insecta	1056	rev2	EF417108
Platycnemis pennipes		Odonata	Insecta	1049	rev2	EF417109
Brachyptera risi		Plecoptera	Insecta	1054		EF417110
Leuctra albida		Plecoptera	Insecta	994	rev2	EF417111
Nemoura cinerea		Plecoptera	Insecta	1032		EF417112
Protonemura intricata		Plecoptera	Insecta	1032		EF417113
Anabolia nervosa		Trichoptera	Insecta	1049	rev2	EF417114
Athripsodes cinereus		Trichoptera	Insecta	1063	rev2	EF417115
Brachycentrus subnubilus		Trichoptera	Insecta	1050	rev2	EF417116
Ceraclea dissimilis	2	Trichoptera	Insecta	1061	rev2	EF417117
Cheumatopsyche lepida	5	Trichoptera	Insecta	1091		EF417118
Cyrnus flavidus	2	Trichoptera	Insecta	1111	rev2	EF417119
Hydropsyche angustipennis		Trichoptera	Insecta	1056		EF417120
Lepidostoma hirtum	2	Trichoptera	Insecta	1041	rev2	EF417121
Molanna angustata		Trichoptera	Insecta	1106		EF417122
Mystacides azurea	1	Trichoptera	Insecta	1111		EF417123
Notidobia ciliaris		Trichoptera	Insecta	1055	rev2	EF417124
Odontocerum albicorne		Trichoptera	Insecta	1119		EF417125
Polycentropus flavomaculatus		Trichoptera	Insecta	1176		EF417126
Potamophylax rotundipennis		Trichoptera	Insecta	1097		EF417127
Psychomyia pusilla	1	Trichoptera	Insecta	1173		EF417128
Musculium lacustre		Bivalvia	Mollusca	986		EF417129
Sphaerium corneum		Bivalvia	Mollusca	986		EF417130

Bathyomphalus contortus		Gastropoda	Mollusca	990		EF417131
Hippeutis complanatus		Gastropoda	Mollusca	1000		EF417132
Physa fontinalis		Gastropoda	Mollusca	990		EF417133
Planorbis carinatus		Gastropoda	Mollusca	1000		EF417134
Potamopyrgus antipodarum	1	Gastropoda	Mollusca	995		EF417135
Radix ovata		Gastropoda	Mollusca	1005		EF417136
Valvata cristata		Gastropoda	Mollusca	1003	rev2	EF417137
Acrobeloides maximus		Nematoda	Nematoda	967		EF417138
Acrobeloides nanus		Nematoda	Nematoda	965		EF417139
Caenorhabditis briggsae		Nematoda	Nematoda	836		EF417140
Caenorhabditis elegans		Nematoda	Nematoda	836		EF417141
Cephalobidae sp. PS1146		Nematoda	Nematoda	965		EF417142
Diplogaster ineritieri		Nematoda	Nematoda	960		EF417143
Panagrolaimus sp. (Brauweiler)	4	Nematoda	Nematoda	955		EF417144
Panagrolaimus sp. (Brombeer Bornheim)		Nematoda	Nematoda	954		EF417145
Panagrolaimus sp. (R18)	6	Nematoda	Nematoda	957		EF417146
Plectus aquatilis	9	Nematoda	Nematoda	975		EF417147
Plectus minimus		Nematoda	Nematoda	977		EF417148
Rhabditis belari	1	Nematoda	Nematoda	791		EF417149
Rhabditis dolichura		Nematoda	Nematoda	807		EF417150
Rhabditis remanei		Nematoda	Nematoda	836		EF417151
Rhabditis terricola		Nematoda	Nematoda	796		EF417152
Romanomermis culicivorax		Nematoda	Nematoda	1017		EF417153
Rhabditidae sp. (*Therimax* ?)		Nematoda	Nematoda	815		EF417154
Alburnoides bipunctatus		Teleostei	Vertebrata	1052		EF417161
Alburnus alburnus		Teleostei	Vertebrata	1052		EF417162
Anguilla anguilla		Teleostei	Vertebrata	1129		EF417155
Pterolebias longipinnis		Teleostei	Vertebrata	1064		EF417191
Aplocheilus panchax		Teleostei	Vertebrata	1051		EF417190
Astyanax sp.		Teleostei	Vertebrata	1068		EF417174
Barbatula barbatula		Teleostei	Vertebrata	1042		EF417163
Barbus barbus		Teleostei	Vertebrata	1063		EF417164
Benitochromis ufermanni		Teleostei	Vertebrata	1059		EF417185
Benthophilus sp.		Teleostei	Vertebrata	1044		EF417173
Brachydanio rerio	1	Teleostei	Vertebrata	1086		EF417169
Brachygalaxias bullocki		Teleostei	Vertebrata	1103		EF417156
Brachygalaxias gothei		Teleostei	Vertebrata	1104		EF417157
Epiplatys bifasciatus	3	Teleostei	Vertebrata	1186		EF417193
Galaxias occidentalis	1	Teleostei	Vertebrata	1139		EF417158
Gasterosteus aculeatus		Teleostei	Vertebrata	1055		EF417178
Gobio gobio		Teleostei	Vertebrata	1082		EF417170
Gymnocephalus cernuus		Teleostei	Vertebrata	1042		EF417181
Hemichromis elongatus		Teleostei	Vertebrata	1051		EF417183
Jordanella floridae		Teleostei	Vertebrata	1044		EF417194
Lepomis gibbosus		Teleostei	Vertebrata	1041		EF417182
Leuciscus cephalus		Teleostei	Vertebrata	1052		EF417165
Leuciscus leuciscus		Teleostei	Vertebrata	1054		EF417166
Marosatherina ladigesi		Teleostei	Vertebrata	1104		EF417188
Misgurnus fossilis		Teleostei	Vertebrata	1050		EF417171
Neogobius syrman		Teleostei	Vertebrata	1044		EF417179
Neolebias kerguennae		Teleostei	Vertebrata	1091		EF417175
Oryzias nigrimas		Teleostei	Vertebrata	1067		EF417186
Pelvicachromis taeniatus		Teleostei	Vertebrata	1059		EF417184
Phoxinus phoxinus	2	Teleostei	Vertebrata	1051		EF417168
Phractura sp.		Teleostei	Vertebrata	1131		EF417176
Plataplochilus aff. ngaensis		Teleostei	Vertebrata	1053		EF417195
Proterhorinus marmoratus		Teleostei	Vertebrata	1044		EF417180
Rivulus xiphidius	6	Teleostei	Vertebrata	1055		EF417192
Rutilus rutilus		Teleostei	Vertebrata	1052		EF417167
Sabanejewia romanica		Teleostei	Vertebrata	1049		EF417172
Salmo salar		Teleostei	Vertebrata	1051		EF417159
Salmo trutta	1	Teleostei	Vertebrata	1051		EF417160
Siluris glanis		Teleostei	Vertebrata	1143		EF417177
Thelmatherina bonti		Teleostei	Vertebrata	1108		EF417189
Xenopoecilus sarasinorum		Teleostei	Vertebrata	1064		EF417187

Sequenced environmental contaminations of DNA samples

Contamination *Acheta domesticus*	8		Hypotrichia	not complete		EF417278
Contamination *Agabus sturmii*			Ciliophora	not complete		EF417279
Contamination *Asellus aquaticus*		96% Vaucheria bursata*	Stramenopiles	not complete		EF417280
Contamination *Cleon dipterum*			Ciliophora	not complete		EF417283
Contamination *Cottus*		93 % Gyrodactylus salaris*	Monogea	not complete		EF417281
Contamination *Habroleptoides confusa*		98% Nitzschia fusiformis*	Stramenopiles	not complete		EF417282
Contamination *Radix ovata*		96% Aspharyngostrigea cornu*	Trematoda, Dige	not complete		EF417284

Remarks:

rev2 = sequenced with rev2, 48 bp shorter as those sequenced with rev1
# = at 3' and 5' differently trimmed than the metazoan dataset
°= single sequences at 3' or 5' end not complete
* = percent sequence similarity according to blast search in GenBank
